# Rapport Building in Written Crisis Services: Qualitative Content Analysis

**DOI:** 10.2196/42049

**Published:** 2024-05-15

**Authors:** Laura Schwab-Reese, Caitlyn Short, Larel Jacobs, Michelle Fingerman

**Affiliations:** 1 Department of Public Health Purdue University West Lafayette, IN United States; 2 Childhelp Scottsdale, AZ United States

**Keywords:** empathy, crisis hotline, child maltreatment, text hotline, chat hotline, telehealth, digital empathy, counseling, child abuse, family violence, crisis, hotline, chat, tele, emotional dynamics, therapeutic relationships, therapy, content analysis, text, inductive, deductive, emotion, affect, emotional dynamic, counseling psychology

## Abstract

**Background:**

Building therapeutic relationships and social presence are challenging in digital services and maybe even more difficult in written services. Despite these difficulties, in-person care may not be feasible or accessible in all situations.

**Objective:**

This study aims to categorize crisis counselors’ efforts to build rapport in written conversations by using deidentified conversation transcripts from the text and chat arms of the National Child Abuse Hotline. Using these categories, we identify the common characteristics of successful conversations. We defined success as conversations where help-seekers reported the hotline was a good way to seek help and that they were a lot more hopeful, a lot more informed, a lot more prepared to address the situation, and experiencing less stress, as reported by help-seekers.

**Methods:**

The sample consisted of transcripts from 314 purposely selected conversations from of the 1153 text and chat conversations during July 2020. Hotline users answered a preconversation survey (ie, demographics) and a postconversation survey (ie, their perceptions of the conversation). We used qualitative content analysis to process the conversations.

**Results:**

Active listening skills, including asking questions, paraphrasing, reflecting feelings, and interpreting situations, were commonly used by counselors. Validation, unconditional positive regard, and evaluation-based language, such as praise and apologies, were also often used. Compared with less successful conversations, successful conversations tended to include fewer statements that attend to the emotional dynamics. There were qualitative differences in how the counselors applied these approaches. Generally, crisis counselors in positive conversations tended to be more specific and tailor their comments to the situation.

**Conclusions:**

Building therapeutic relationships and social presence are essential to digital interventions involving mental health professionals. Prior research demonstrates that they can be challenging to develop in written conversations. Our work demonstrates characteristics associated with successful conversations that could be adopted in other written help-seeking interventions.

## Introduction

### Background

Since the 1990s, mental health providers have explored how to support clients via internet-based communication [1]. Prior work suggests that young people may be particularly interested in these approaches, as digital communication feels more private and emotionally safe [2]. However, internet-based communication, particularly written communication, may have significant barriers for providers and clients, including the inability to express emotion and challenges in communicating clearly [2]. Currently, there is limited evidence on how to overcome these communication issues in counseling settings [3]. Understanding how to do so may help telehealth providers build stronger therapeutic relationships, thus improving the help-seeking process. Further, this understanding may help agencies improve services and training for providers.

### Technology-Based Mental Health Interventions

Technology-based (ie, telehealth) mental health services may not be as effective as in-person services. One recent meta-analysis suggests that videoconferencing-based therapeutic relationships may be inferior to those developed during in-person therapy [4]. However, there may be times when in-person care is not accessible or feasible. Nearly half of people in the United States live in a mental health shortage area, and there are areas with less than 2 psychiatrists per 100,000 residents [5,6]. As a result, increasing access to mental health care may depend on telehealth approaches. Within telehealth studies, interventions retaining elements of human contact are more effective than entirely computer-based interventions [7,8].

Two critical aspects of the helping relationship, therapeutic relationship and social presence, may be challenged when engaging digitally. A therapeutic relationship based on mutual trust, respect, empathy, and positive regard is essential in counseling [9]. Hundreds of studies have confirmed the importance of this collaborative relationship [10]. For most help-seekers, confidence in the provider, including perceptions of empathy and expertise, is key to developing a strong relationship [11-13].

Social presence [14], the sense of connecting and being with another, is another element that may be compromised during digital communication. Social presence may also be defined as the degree to which the other person feels “real” [15]. Although it is a natural element of face-to-face counseling, telehealth providers may have to be intentional in building a social presence. When conversing with unknown entities through written technology, it is common to question whether the other person is a human or a bot [16], in part because people are not reliably able to differentiate between the two [17]. Some prior work suggests that social presence is an important aspect of digital helping relationships because it assists in building therapeutic partnerships, professional bonds, and open communication [18,19].

Much of the literature on telehealth counseling focuses on verbal communication via videoconferencing or phone [20]. Few studies examine written mental health counseling services, and there is reason to believe that spoken and written communication are substantially different. In a recent review of the digital therapeutic relationship, Bantjes and Slabbert [20] suggest practical strategies for establishing rapport in digital spaces, such as maintaining eye contact, having high-speed internet to avoid lags, and attending to lighting and microphone placement. These strategies improve the audio and visual cues, which are not applicable to written communication.

### Written Interventions

The literature on written counseling is limited [21,22]. In the 1990s, a small group of mental health providers offered therapy via email [23]. This early work identified several possible strengths and limitations. It was helpful for clients to write about their feelings, and the anonymity of email allowed them to share more openly. This asynchronous approach also increased many individuals’ sense of control, as they could choose when and where to engage with the therapist. Conversely, building a relationship and understanding nuances could be difficult without the usual social cues [23]. Two more recent literature reviews support many of the impressions formed by the early adopters, although most of the studies had very small sample sizes [22,24].

Written interventions may be challenging for the provider and patient, and both experience similar challenges. One randomized controlled trial of a chat-based cognitive behavioral therapy demonstrated reduced depression symptoms after 10 sessions [25]. In a parallel qualitative study, participants reported mixed perceptions of the experience [26]. Some reported feeling more able to share openly and process because of the anonymous platform. Others felt it was challenging to develop a relationship and express complex feelings and thoughts via writing [26]. Another study assessed differences between email-based cognitive behavioral therapy and unguided treatment. The email and unguided programs had better outcomes than the wait-list control group for some, but not all, outcomes [27,28]. Other studies, with and without in-person or telephone comparison groups, showed similarly mixed results [29,30]. In one unpublished dissertation, counselors who provided email services reported feeling substantial anxiety due to uncertainty, limited sensory information, and concerns about misunderstanding clients’ intentions [31]. The lack of visual, verbal, and social cues was particularly challenging [31]. They often focused more on the tasks and transactional aspects of helping to manage these uncertain dynamics [31]. Many also talked about needing much more time than usual to build the therapeutic relationship, although it did eventually happen for most [31].

Beyond mental health counseling, some recent work has examined written communication for brief counseling and advocacy [3,32,33]. Overall, privacy, autonomy, control, anonymity, and accessibility are seen as benefits of written services [34-36]. Building social presence and connection is an important aspect of the experience [34]. Often this professional connection builds over time, but because the help-seeker and crisis counselor or advocate do not have an ongoing relationship, it may be particularly difficult to communicate adequately and build a relationship [2,30,37,38]. Correctly understanding sarcasm, humor, and other nuanced language is particularly challenging in these brief interventions [3,39]. Like mental health counseling, the impact of written crisis and advocacy services is unclear in the current literature and may depend on geographical location, counselor training, and the help-seekers’ situations [33,40-46].

### Study Purpose

Overall, establishing a human connection based on a strong therapeutic relationship and social presence will likely result in more effective, acceptable interventions. Providing crisis services is complex, and the confines of written communication create additional challenges. Rapport-building is particularly difficult, and mistakes may cause the help-seeker to feel worse [47]. However, there are not yet best practices for building rapport in these conversations, as existing approaches to rapport-building often depend on verbal and nonverbal cues [48]. As part of a larger study focused on building best practices for written hotlines, we worked with a child maltreatment-focused text or chat hotline. This analysis aims to categorize crisis counselors’ efforts to build rapport and convey active listening in written conversations. Using these categories, we identified characteristics associated with successful conversations, as reported by help-seekers. This work provides an important foundation for how to build therapeutic relationships in written mental health and hotline services.

## Methods

### Data Source

The data for this study are from the PACTECH (Prevent Abuse of Children Text and Chat Hotline), the text and chat arm of the Childhelp National Child Abuse Hotline [49]. Since 1982, Childhelp has offered 24/7 phone-based hotline services focused on support and resources related to child maltreatment. In 2018, the hotline expanded to include text and chat capabilities. Crisis counselors are employees rather than volunteers. Most are master-level professionals with specialized training in hotline services and child maltreatment. After conducting a quantitative pilot evaluation for 2 years, hotline leadership partnered with the lead author to use qualitative and mixed method approaches to identify best practices for services. As part of the data sharing agreement, the lead author and her research team received access to deidentified transcripts and metadata from conversations that were purposefully selected to represent a wide range of experiences and perceived outcomes.

### Ethical Considerations

The Purdue University Institutional Review Board approved the research protocol (IRB-2020-965). The service terms and conditions disclosed that data may be shared with researchers. As a secondary data analysis of deidentified data, additional consent from participants was not required by the Institutional Review Board. The contract teams from Purdue University and Childhelp negotiated the terms of the data sharing agreement, including data security and access. As a result of the data sharing agreement, the data may not be released publicly.

### Sample

The sample consists of 314 purposely selected conversations out of the 1153 text and chat conversations during July 2020. In addition to maintaining the written transcript of the conversation for 60 days, Childhelp collects preconversation and postconversation surveys from the help-seekers. The preconversation surveys focus on help-seeker characteristics (ie, age, gender, state of residence, and referral source), while the postconversation survey focuses on their perceptions of the conversation (eg, do they feel more hopeful, less stressed, and more prepared). We used maximum variation sampling to capture diverse help-seekers and outcomes, although not necessarily in the proportions present in the overall data [50]. This approach is particularly useful when looking for diverse perspectives, as was the case for our study. We sampled based on the preconversation and postconversation surveys. In our sample, 297 (94.6%) help-seekers answered at least 1 presurvey question, and 263 (83.8%) answered at least 1 postconversation survey question. First, we selected conversations where help-seekers reported that they were satisfied, unsatisfied, or mixed. We also included some conversations without surveys to reduce survey response bias. Then, we reviewed the demographic characteristics of the selected conversations to ensure help-seekers of different ages, races or ethnicities, and genders were included in the sample. For example, most help-seekers are girls, so there were relatively few conversations with boys in our initial sample. We added additional conversations with boys to ensure the results were not only relevant to girls.

We analyzed and reported the findings from all 314 conversations. When reporting quotes, however, we were particularly interested in the 45 conversations where help-seekers reported in the postconversation survey that the hotline was a good way to seek help and that they were a lot more hopeful, a lot more informed, a lot more prepared to address the situation, and experiencing less stress. Except when specifically referencing less successful conversations, all example quotes come from these conversations, as they represent those most successful from the help-seekers’ perspectives. All quotes are reported verbatim from the conversations, including any errors.

### Analysis

We used qualitative content analysis to process the conversations. We used both inductive and deductive processes to develop the codes. The first draft of the coding frame was based on our work with child maltreatment–related conversations within the Crisis Text Line [51-53]. Then, we revised the framework based on the content of the conversations.

Our development process followed the adaptation of grounded theory described by Schreier [54]. The lead author and her graduate research assistant reviewed all the conversations. During a second review of the conversations, we took notes on commonalities within the conversations, emphasizing material not captured in the first draft of the codebook. As we refined the codebook, all team members met weekly to discuss emerging materials and define and develop codes. After completing the framework and definitions, we coded 30 conversations and met to compare the code applications. We discussed differences in coding and refined the framework with the entire team. Then, we coded 30 additional conversations and assessed the coder agreement. After the second round of pilot coding, we reached 95% agreement on the codes and moved to code the full data set. In sum, we had 127 codes in the codebook, which were applied 22,326 times. After coding all the conversations, we reviewed the materials within each code. This process followed the segmentation process described by Schreier [54], where coded materials are decontextualized and reviewed to identify commonalities and themes. Through this process, we also assessed whether we met saturation, which occurs when all categories have been identified in the data set. Schrier’s [54] definition of qualitative content analysis saturation is different from other forms of qualitative methods. In other forms of qualitative analysis, saturation refers to the point at which reviewing additional material does not provide new information. We informally assessed this type of saturation by examining whether all codes were used if we considered only half of the sample. We found that all codes were used when we reviewed 2 different randomly selected split samples, which suggests that few new insights would be gained if we added additional conversations to our sample. After conducting these checks, we categorized the conversations by the outcomes and focused on similarities and differences across the groups.

For this analysis, we focused on the codes related to rapport building and active listening conversations. There were two main types of approaches used by crisis counselors: (1) counseling approaches and (2) evaluation-based language. Active listening skills, otherwise known as attending skills, are how counselors build connections with clients, express empathy, and convey that they are listening [48]. These skills may be defined slightly differently; asking questions, paraphrasing, reflecting feelings, and interpreting or summarizing the situation are generally recognized skills. We added validation [55] and unconditional positive regard [56], which are also commonly incorporated into helping relationships. Evaluation-based language, such as praise and apologies, is commonly used by adults when talking with children [57]. These statements differ from other approaches because the counselor’s evaluation of the situation is included.

We also examined how these approaches differed between the help-seekers most satisfied with the conversation (ie, answered all after-conversation survey questions as “Yes”) and those who were least satisfied with the conversation. We intended to define the least satisfied as those who answered all the after-conversation survey questions as “No.” However, only 4 people fit that criterion, so we included all help-seekers who answered most of the questions negatively.

### Research Team

The research team included the lead author, a graduate research assistant, and 2 collaborators at Childhelp. The lead author is a family violence prevention researcher with a PhD in public health and an MA in counseling. She has experience conducting qualitative analyses of written hotline transcripts. The graduate research assistant was a master of public health student and had worked on the lead author’s research team for 3 years. She had experience with qualitative child maltreatment research. The Childhelp collaborators have substantial experience in hotline counseling and leadership. One has an MS in counseling psychology. The second has an MS in family and human development and an MEd in guidance counseling.

## Results

### Help-Seeker Characteristics

Overall, our sample of help-seekers was generally similar to Childhelp’s overall text and chat users (Table 1) [58,59]. Help-seekers tended to be female, young, and seeking help for themselves. Overall, they were generally at least a little more hopeful, informed, and prepared to deal with the situation after the conversation (Table 2).

**Table 1:** Characteristics of help-seekers (n=314).

Characteristics			Help-seekers, n (%)
**Gender**			
	Female	220 (70.1)	
	Man	53 (16.9)	
	Gender expansive	22 (7)	
	No response	19 (6)	
**Age (years)**			
	10-11	9 (2.9)	
	12-13	45 (14.3)	
	14-15	80 (25.5)	
	16-17	61 (19.4)	
	18-24	35 (11.2)	
	≥25	52 (16.6)	
	No Response	32 (10.2)	
**Relationship to maltreated child**			
	Self^a^	172 (54.8)	
	Family	40 (12.7)	
	Friend	37 (11.8)	
	Other adult	17 (5.4)	
	Unknown to child	22 (7)	
	Other	26 (8.3)	

^a^Includes children who were distressed but did not necessarily describe events consistent with maltreatment.

**Table 2:** Help-seekers’ perceptions of conversations (n=314).

Perceptions			Help-seekers, n (%)
**More positive or hopeful^a^**			
	A lot	106 (33.8)	
	A little	121 (38.5)	
	Not at all	36 (11.5)	
	No response	51 (16.2)	
**More informed^b^**			
	A lot	154 (49)	
	A little	84 (26.8)	
	Not at all	21 (6.7)	
	No response	55 (17.5)	
**More prepared^c^**			
	A lot	93 (29.6)	
	A little	112 (35.7)	
	Not at all	45 (14.3)	
	No response	64 (20.4)	
**Less stressed^d^**			
	Yes	120 (38.2)	
	Maybe	60 (19.1)	
	No	71 (22.6)	
	No response	63 (20.1)	
**Good approach^e^**			
	Yes	205 (65.3)	
	Maybe	39 (12.4)	
	No	18 (5.7)	
	No response	52 (16.6)	

^a^Do you feel more positive or hopeful after this chat/text session?

^b^Did you get the information you needed from this chat or text session?

^c^Do you feel better prepared to deal with the situation after this chat or text session?

^d^Do you feel less stress after the chat or text session?

^e^Was using chat or text a good way for you to get help?

### Active Listening Skills

#### Paraphrasing Information and Feelings

When paraphrasing (387 times across 170 conversations), the crisis counselor repeated what was said by the help-seeker in a way that honed the focus of the conversation. Often, it included the most important words shared by the help-seeker, along with a shortened, clarified version of the essential information or feelings. For example, when seeking to understand the situation, a crisis counselor said, “It does not sound like she is able to listen to your needs and wants at this time.” At other times, the crisis counselor wanted to convey that they have been listening. Saying, “...you mentioned that they are screaming at him and from what you have said it sounds like they might be being really aggressive with him” demonstrated that they have been paying attention to the information shared.

Sometimes, the crisis counselors reflected the feelings shared by the help-seeker, saying things like, “That sounds like it can be frustrating from what you shared,” “it sounds very overwhelming and scary,” or “I can see how stressful this is.” In these situations, the crisis counselor was often distilling the feelings to support the help-seeker in identifying what is most bothering them about the situation or what feeling is driving their response to the situation. Once the help-seeker recognized the most troubling aspect of the situation, they were often more able to brainstorm ways to address it with the crisis counselor.

#### Interpretation

Interpreting the situation was also common (236 times across 125 conversations). Often, help-seekers were confused or had ambivalent thoughts about the situation. In these cases, they usually struggled to identify the next steps or reduce their emotional activation. By interpreting the situation, the crisis counselors offered a coherent overview of the situation and a different perspective. In most active listening skills, crisis counselors stayed quite close to the information provided by the help-seeker (eg, paraphrasing or reflecting what was said). When interpreting the situation, crisis counselors often included their perspectives on the situation with the intent of supporting the help-seeker to see themes or new ideas. For example, one help-seeker shared that their caregivers regularly say hurtful things about their gender identity and sexual orientation, scream and yell, and tell the help-seeker that they are a disappointment. In response, the crisis counselor said, “Sounds like it would be very hard to be happy living with people who treat you like that.” Although the help-seeker had not overtly shared about their unhappiness, this interpretation led to the help-seeker sharing about active suicidal ideation.

#### Open Questions

Open questions (208 across 124 conversations) served multiple purposes. At the beginning of the conversations, they invited the help-seeker to share about the experience. For example, “Could you tell me what’s going on?” or “What’s making you feel unsafe?” was used to begin the conversation in a nonthreatening way. As the conversation moved to explore the issues, open questions could elicit specific details (eg, “What’s happened since then?” and “What does that mean?”) or focus attention on feelings (eg, “How does it make you feel when your mom lashes out?” and “How are you feeling about all this happening?”).

### Other Common Counseling Approaches

#### Validation

Validation was the most used approach to active listening (647 times across 226 conversations), and it took many forms depending on the situation. Throughout the conversations, it was used to affirm the help-seeker, their feelings, and their thoughts. For example, one counselor said, “It can be hard living in a house where you don’t feel supported and respected.” In this situation, the help-seeker had a difficult relationship with a father, who regularly called the help-seeker “overdramatic or a crybaby.” By validating the difficulty of feeling unsupported, the crisis counselor communicated that the help-seeker and their feelings were important.

In other instances, the crisis counselor validated the help-seeker’s perspectives about what was or was not appropriate behavior within families. In one instance, a help-seeker shared concerns about an older sibling’s treatment of an infant. The brother was rough with the infant and burned the infant with hot milk. In response, the crisis counselor said, “I can see why you would be concerned for the baby’s safety.” In doing so, the crisis counselor communicated that the help-seeker’s feelings were valid but without confirming that the infant was being maltreated. The crisis counselor had not seen evidence of the situation, so they could not accurately validate whether the infant was being maltreated. Simple phrases, such as “I hear you. This is difficult,” “That must be really hard for you,” and “It’s okay to feel stressed that is normal,” also validated the help-seeker and their perspectives.

#### Unconditional Positive Regard

Unconditional positive regard (102 times across 66 conversations) occurred when crisis counselors provided basic acceptance and support of the help-seeker, regardless of their behavior or things that have been done to them. Unconditional positive regard primarily focused on the abuse experience. It was common for counselors to say things like, “No one deserves to be abused” or “No one deserves to be treated like that.” These statements were often particularly well received by help-seekers, like this example:

You don’t deserve to be emotionally abused. It’s not o.k.

Counselor

Thank you for saying that. You are the first person ive ever talked about this personally with.

Help-seeker

### Evaluation-Based Language

#### Overview

Evaluation-based language involved a judgment by the crisis counselor about whether an aspect of the help-seekers’ experiences was good (eg, behavior worthy of praise) or bad (eg, an apology for something that happened to the help-seeker). Evaluation and judgment are generally not a part of helping relationships [48,60,61] but are quite common when adults speak with children [57,62]. Although these approaches are not generally part of counseling relationships, there is nothing inherently wrong with using them intentionally.

#### Praise

Praise (268 times across 145 conversations) occurred when the crisis counselor conveyed that they approved of the help-seekers or their behavior. Sometimes, praise focused on the behaviors occurring during the conversations, like “Thank you for sharing with me” and “I’m glad you reached out today.” At other times, the praise centered on behaviors that they would do in the future, such as “Yes, I believe you’re doing the right thing by calling)” and “I think that will be a good move for you.”

#### Apologies

Apologies (372 times across 213 conversations) tended to focus on the help-seeker’s situation or issues with the hotline. Apologies for the hotline were usually about a technical difficulty (eg, “sorry, our system is not working well”). Apologies about the help-seeker’s situation could be very broad, such as “I’m so sorry to hear about all of this” and “I’m so sorry that you’re having to go through this.” Apologies could also be specific to the situation, like “I am sorry to hear Mom yelled at you yesterday too.”

### Differences Between Successful and Less Successful Conversations

There were some differences in active listening skills, other counseling skills, and evaluation-based language between successful and less successful conversations. Although the sample of successful and less successful conversations was too small for formal statistical analysis, some commonalities emerged. First, although conversations were approximately the same length, less successful conversations tended to have more statements that attended to rapport building. Second, there were also differences in how the counselors applied these approaches. Unlike the preceding sections, this section includes quotes from both successful and less successful conversations.

Overall, counselors in less successful conversations tended to be vague or to directly repeat what was said by the help-seeker. These differences were particularly apparent when counselors were paraphrasing, asking open questions, or apologizing. For example, paraphrasing in less successful conversations tended to be either very vague (eg, “It sounds like you are being hurt already”) or very specific (eg, “I am hearing you have some future plans to get a job and earn your own money...”). In the last example, the help-seeker used the same phrasing in their previous statement. Conversely, successful conversations tended to be specific without direct repetition (eg, “Sounds like they are something to help you cope”). Similarly, less successful conversations tended to include open questions that were either broad (eg, “What’s happening?”) or focused on clarifying how the crisis counselor could help (eg, “How are you hoping that I can help?”). Some successful conversations also included questions clarifying how the crisis counselor could help, but it was more common to ask more specific questions, like “What is it that you would like to vent about?” and “What are their thoughts on CPS involvement?” Finally, crisis counselors used generally vague apologies about the situation in less successful conversations. Saying things like “I am so sorry this happened to you” or “I’m sorry to hear that” was common. Although some successful conversations also included these types of apologies, it was more common to pair the apology with a specific reason, such as “I’m so sorry that you have been experiencing this for so long” or “I am sorry to hear Mama is sick.”

## Discussion

### Principal Results

Overall, our study suggests that it is possible to build therapeutic relationships via a text and chat hotline with individuals seeking child maltreatment–related information and support. Approximately 15% (n=45) of our sample reported that the hotline was a good way to seek help and that they were a lot more hopeful, a lot more informed, a lot more prepared to address the situation, and experiencing less stress. However, our sample was intentionally selected to represent a wide range of help-seeker perceptions, so this does not indicate that 15% of the hotline’s help-seekers felt this way. Based on the 2022 Childhelp data report, about 85% of help-seekers reported getting the information they needed, 80% of help-seekers reported feeling more hopeful after the conversation, and 75% reported feeling better prepared to deal with the situation [59]. These percentages suggest that the hotline provides a well-received service.

Generally, counselors built rapport through active listening skills, other counseling techniques, and evaluation-based language (ie, apologies, praise). Through active listening skills and other counseling techniques, counselors often expressed that they were listening, wanted to understand the help-seekers, and cared for them. They expressed their approval or disapproval of the help-seekers and aspects of their experiences through evaluation-based language. Although there is nothing inherently wrong with using apologies and praise, they tend to be avoided in many therapeutic approaches. Praise may undermine intrinsic motivation (ie, internal drive) and reduce engagement in the process [63-65]. Further, these types of evaluation-based language are rooted in control, as they are given based on something that another individual (ie, the crisis counselor) deems desirable [63]. As a result, the help-seeker might seek praise by giving answers that they believe the crisis counselor wants to receive instead of accurate answers, which may reduce the benefit of the conversation. However, praise and compliments may be a quick way to build encouraging feelings [66]. As it is challenging to build relationships via writing, praise may be one way to build a relationship quickly. Additional research into the impact of evaluation-based language is necessary to understand its role in written crisis counseling.

There were some differences between successful and less successful conversations. Surprisingly, less successful conversations tended to include more attending language than successful conversations. However, there were differences in the ways that crisis counselors apply these techniques. Overall, the crisis counselors in successful conversations tended to be more specific and tailor their responses to the help-seekers. Possibly, counselors who gave tailored responses built rapport more quickly; thus, fewer attending statements were required. If this is the case, they could move to problem-solving more quickly, which may also contribute to help-seekers’ perceptions that they were more prepared to address the situation and were more informed. These tailored responses may increase help-seekers’ perceptions that the crisis counselor is invested in the conversation. Several help-seekers explicitly asked if they were speaking with a bot in this sample. Having tailored responses may increase crisis counselors’ social presence and reduce help-seekers’ concerns about whether they are “real.” As organizations consider using large language models and chatbots in these types of services, careful attention should be given to help-seekers’ perceptions about the service and its appropriateness for the audience. As the National Eating Disorders Association learned when its wellness chatbot began providing diet information, large language models trained on outside data may not be a good fit for conversations with help-seekers [67].

### Limitations

Our work has several limitations, including some inherent to secondary data analysis. First, we could not speak with the help-seekers or the counselors about the conversations. Although we were able to identify similarities across well-received conversations, it is possible that other aspects of the conversations contributed to help-seekers’ perceptions. Second, we do not know how these conversations shaped long-term outcomes. Moreover, it is difficult to follow up with help-seekers, as evidenced by the 6% response rate to a 2-week follow-up survey conducted by the National Domestic Violence Hotline [68]. Further, many of the help-seekers in this sample indicated that it is unsafe to speak aloud about their experiences, so qualitative data collection with this sample would likely have an even lower response rate. It would be more feasible to speak with counselors about their experiences, but their perspectives may be disconnected from those of the help-seekers. Despite this limitation, we incorporated the help-seekers’ perspectives through the postconversation survey, which is more than is usually possible in secondary data analysis.

Our work may not generalize to conversations unrelated to child maltreatment. As a child maltreatment–specific hotline, all conversations included elements of child maltreatment. Conversations about other topics may require other approaches. However, our results are consistent with prior work on building rapport in other forms of counseling [48,55,56], so it is reasonable to expect these findings would translate to written conversations about other topics.

### Comparison With Prior Work

To the best of our knowledge, there is no other work examining specific ways to build a therapeutic relationship in written mental health counseling or crisis counseling. However, the ways that crisis counselors attended to the dynamics of the conversations were generally like those found in in-person counseling [48,55,56].

Telehealth approaches to counseling may be particularly important for young people experiencing maltreatment. Other formal resources, such as law enforcement, schools, and child protection systems, often fail to respond adequately [53,69,70]. Further, internet-based approaches, particularly written approaches, are highly acceptable to young people experiencing maltreatment [69]. In our sample and past research, children shared that they could not call resources because an audible conversation would cause parents to know they were seeking help. In work conducted with Crisis Text Line, it was common for young people sharing child maltreatment to report that the abuse escalated when parents discovered their attempts to seek help. Written, anonymous communication that is available 24/7 may be a safer way for these young people to seek help. Thus, written communication may be particularly important for children in unsafe homes.

There is also limited evidence on how to respond when young people share maltreatment experiences. Regardless of the ability to impact or end the maltreatment, individuals who receive a child maltreatment disclosure need to receive an appropriate, supportive response [71-74]. Supportive responses encourage the young person experiencing maltreatment to reframe their experience, which substantially reduces the likelihood of poor outcomes otherwise associated with maltreatment [75]. Conversely, unsupportive experiences often have long-lasting consequences [74,76]. Receiving a hurtful or unsupportive response increases the likelihood that the young person will experience more significant physical and mental health issues [74,76,77]. Unfortunately, many young people receive unsupportive responses to their disclosures [53,70]. Often, they report that others, particularly adults, do not believe them and are unwilling to help [70,78]. These experiences reduce their willingness to seek help or share their experiences in the future [70]. Our work suggests that responding to these disclosures adequately in written conversations is possible.

Our work also contributes to a small body of literature on using text and chat hotlines to provide services to people experiencing violence more generally. Michigan State University added chat services to its existing sexual assault support and advocacy hotline. Their evaluation was consistent with many of the benefits and limitations of other forms of written counseling, including challenges with nuance, misunderstanding written language, and communicating empathy [3]. However, the format also gave help-seekers a greater sense of control [3]. Another study focused on agencies providing digital violence-related support and advocacy services [32]. This work also emphasized the importance of clear communication and building rapport, although help-seeker perceptions of these factors were not assessed [32].

### Conclusions

Building therapeutic relationships and social presence are important components of digital interventions involving mental health professionals. Prior research suggests that they can be challenging to develop in written conversations. Our work demonstrates characteristics of conversations associated with greater satisfaction among help-seekers. These findings may be adopted by other organizations building mental health or support interventions that include written communication. However, additional research is needed to identify how to train providers to adopt these strategies while also tailoring their approach to the help-seeker. Further, our findings may inform future work with large language models, including how large language models could contribute to these interventions. However, future research is needed to understand how help-seekers would interface with these methods and to ensure that the models consistently convey appropriate, supportive information.
